# A comprehensive evaluation of early potential risk factors for disease aggravation in patients with COVID-19

**DOI:** 10.1038/s41598-021-87413-6

**Published:** 2021-04-13

**Authors:** Qiang Tang, Yanwei Liu, Yingfeng Fu, Ziyang Di, Kailiang Xu, Bo Tang, Hui Wu, Maojun Di

**Affiliations:** 1 Department of General Surgery, Shiyan Taihe Hospital, Hubei University of Medicine, Shiyan, Hubei China; 2 Department of Urology, Jingzhou Central Hospital, The Second Clinical Medical College, Yangtze University, Jingzhou, China; 3 School of Public Health, Xinxiang Medical University, Henan, China

**Keywords:** Microbiology, Risk factors

## Abstract

The 2019 Coronavirus Disease (COVID-19) has become an unprecedented public crisis. We retrospectively investigated the clinical data of 197 COVID-19 patients and identified 88 patients as disease aggravation cases. Compared with patients without disease aggravation, the aggravation cases had more comorbidities, including hypertension (25.9%) and diabetes (20.8%), and presented with dyspnoea (23.4%), neutrophilia (31.5%), and lymphocytopenia (46.7%). These patients were more prone to develop organ damage in liver, kidney, and heart (P < 0.05). A multivariable regression analysis showed that advanced age, comorbidities, dyspnea, lymphopenia, and elevated levels of Fbg, CTnI, IL-6, and serum ferritin were significant predictors of disease aggravation. Further, we performed a Kaplan–Meier analysis to evaluate the prognosis of COVID-19 patients, which suggested that 64.9% of the patients had not experienced ICU transfers and survival from the hospital.

## Introduction

In December 2019, an outbreak of unidentified pneumonia aroused great attention throughout the world and is considered to be not only a pandemic, but also a disaster. On January 27, 2020, the World Health Organization (WHO) issued worldwide surveillance and vigilance recommendations of highly contagious respiratory diseases^1^. Sequence analyses of coronaviruses from lower respiratory tract samples have shown a structure typical of other coronaviruses, such as SARS and MERS coronaviruses. Afterwards, the WHO named the unidentified coronavirus SARS-CoV-2^2,3^. SARS-CoV-2 belongs to the clade of the *sarbecovirus* subgenus of the *Orthocoronavirinae* subfamily. Evidence has been found that SARS-CoV-2 is extremely contagious to humans and can be transmitted through respiratory droplets, contact, and even via faecal-oral transmission^4^.

As has been reported in the literature, patients with COVID-19 mainly presented with fever, cough, fatigue, myalgia, and dyspnoea. Several studies have shown that the majority of patients are considered to have a favourable prognosis; however, elderly men and those with underlying diseases, including hypertension, diabetes, and chronic obstructive pulmonary disease (COPD), have a higher risk of developing acute respiratory distress syndrome (ARDS), which may be the leading cause of death^5^. Therefore, an investigation of the risk factors that are associated with disease aggravation is greatly warranted. In this study, we investigated the clinical characteristics and relevant factors that are associated with the outcomes of patients with COVID-19 infections, which may provide considerable value for the early identification of individuals who are at risk of disease aggravation and who are most likely to benefit from intensive care treatment.

## Results

### Demographic and clinical characteristics

**Figure 1:**
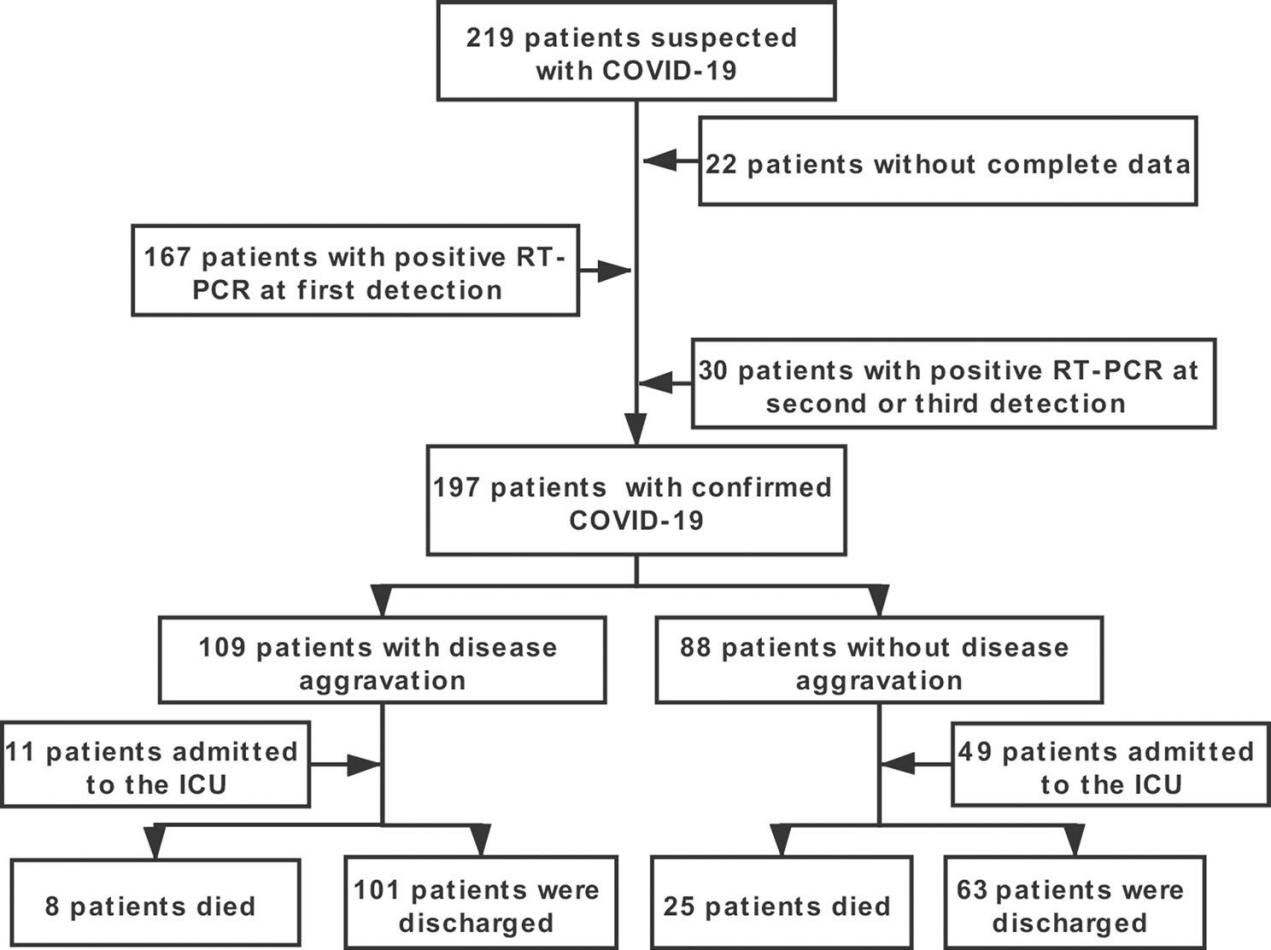
Study flow of patient selection, classification, identification and outcomes in our study.

In this study, a total of 197 patients with COVID-19 were confirmed from the designated hospital, and the study follow diagram were shown in Fig. 1. The demographic and clinical characteristics are shown in Table 1. The median age of the patients was 66.5 years (IQR: 7–76), and 97 (49.2%) patients were older than 60 years. Additionally, 120 patients were males. In addition, 18.3% of the patients had smoking histories. Seventy-two (36.5%) patients had at least one underlying chronic disease, including hypertension (25.9%), diabetes (20.8%), COPD (18.8%), cardiovascular disease (11.2%), cerebrovascular diseases (9.1%), malignancy (8.1%), chronic kidney disease (7.6%), chronic liver disease (10.2%), and HIV infection (4.6%). Among the patients of the two groups, the disease aggravation patients were older and had more comorbidities (hypertension, diabetes, or COPD). Furthermore, COVID-19 patients with ACEI/ARB therapy were enrolled, and we found that approximately 50.7% (29/57) of the patients had received ACEI/ARB therapy in the aggravation group, which was much higher than that of the control group patients (25.7%).

**Table 1:** Baseline characteristics of patients infected with 2019-nCoV.

Clinical characteristics	Total patients (n = 197)	Non-aggravation (n = 109)	Aggravation (n = 88)	p value
Age (year, IQR)	66.5 (7–76)	53 (32–64)	60 (47.5–67)	< 0.001
Male (n/%)	120 (60.9%)	54 (49.5%)	66 (75.0%)	< 0.001
Medical workers (n/%)	36 (18.3%)	33 (30.3%)	3 (3.40%)	< 0.001
Smoking history (n/%)	46 (23.4%)	19 (17.4%)	27 (30.7%)	0.03
**Comorbidity (n/%)**				
Hypertension	51 (25.9%)	21 (19.3%)	30 (34.1%)	0.02
Diabetes	41 (20.8%)	14 (12.8%)	27 (30.7%)	0.003
ACEI or ARB (n = 92)	38/92 (41.3%)	9/35 (25.7%)	29/57 (50.7%)	0.017
COPD	37 (18.8%)	13 (11.9%)	24 (27.3%)	0.006
Cardiovascular disease	22 (11.2%)	9 (8.3%)	13 (14.8%)	0.15
Cerebrovascular disease	18 (9.1%)	7 (6.4%)	11 (12.5%)	0.15
Cancer	16 (8.1%)	10 (9.2%)	6 (6.8%)	0.55
Chronic liver disease	20 (10.2%)	9 (8.3%)	11 (12.5%)	0.33
Chronic kidney disease	15 (7.6%)	9 (8.3%)	6 (6.8%)	0.71
HIV infection	9 (4.6%)	4 (3.7%)	5 (5.7%)	0.52

Data are median (IQR), n (%).

The symptom onset of the patients is shown in Table 2. The most common symptoms at the onset of illness were fever (80.7%) and dry cough (79.7%), followed by fatigue (63.5%), chest distress (38.6%), and dyspnoea (23.4%). The majority of patients (76.6%) had both fever and cough. Eighty-nine (45.3%) patients had a fever with fatigue, and 72 (36.8%) patients had a fever with dyspnoea. The less common symptoms were rhinorrhoea (4.6%), runny nose (4.6%), and chest pain (7.6%). Notably, we found that 24 patients presented with at least one gastrointestinal symptom, including abdominal pain (9.6%) and nausea or vomiting (11.2%). In addition, 11 (5.6%) patients were clinically characterized by having conjunctival hyperaemia. Although these digestive and ocular symptoms were less common in COVID-19 patients, special attention should be paid to the care of this unique group of patients. The median time from symptom onset to hospital admission was 5.0 (3–8) days. Compared with patients without disease aggravation, the disease aggravation patients presented with a higher percentage of chest stress and dyspnoea, as well as decreased oxygen partial pressure.

**Table 2:** Signs and symptoms of the two groups.

Clinical characteristics	Total patients (n = 197)	Non-aggravation (n = 109)	Aggravation (n = 88)	p value
**Signs and symptoms at admission (n/%)**				
Fever	159 (80.7%)	89 (81.7%)	70 (79.5%)	0.71
Dry cough	157 (79.7%)	88 (80.7%)	69 (78.4%)	0.69
Productive cough	40 (20.3%)	18 (16.5%)	22 (25.0%)	0.14
Nasal congestion	15 (7.6%)	10 (9.2%)	5 (5.7%)	0.36
Rhinorrhea	9 (4.6%)	6 (5.5%)	3 (3.4%)	0.73
Myalgia or arthralgia	45 (22.8%)	21 (19.3%)	24 (27.3%)	0.18
Headache and dizziness	29 (14.7%)	16 (14.7%)	13 (14.8%	0.99
Runny nose	9 (4.6%)	6 (5.5%)	3 (3.4%)	0.48
Fatigue	125 (63.5%)	63 (57.8%)	62 (70.5%)	0.73
Chest distress	76 (38.6%)	34 (31.2%)	42 (47.7%)	0.02
Chest pain	15 (7.6%)	6 (5.5%)	9 (10.2%)	0.21
Chills	23 (11.7%)	10 (9.2%)	13 (14.8%)	0.22
Sneeze	8 (4.1%)	5 (4.6%)	3 (3.4%)	0.73
Dyspnea	46 (23.4%)	16 (14.7%)	30 (34.1%)	0.001
Abdominal pain	19 (9.6%)	9 (8.3%)	10 (11.4%)	0.46
Nausea or vomiting	22 (11.2%)	12 (11.0%)	10 (11.4%)	0.94
Conjunctival hyperemia	11 (5.6%)	5 (4.6%)	6 (6.8%)	0.54
Oxygen partial pressure (%)	94 (87–97)	97 (94–99)	88 (80–93)	< 0.001
**Onset of symptom to hospital admission (day)**	5(3–8)	5 (3–9)	5 (3–7)	0.40

Data are median (IQR), n (%).

### Laboratory indices

The laboratory findings of the confirmed patients are summarized in Table 3. Of the 197 patients, 62 (31.5%) cases showed an increased number of neutrophils, 92 (46.7%) cases had lymphocytopenia, and 37 (18.8%) cases developed thrombocytopenia. Severe patients showed liver injury with elevated levels of aspartate aminotransferase (AST: 34%), alanine aminotransferase (ALT: 36.5%), total bilirubin (TBIL: 7.6%), direct bilirubin (DBIL: 12.2%), and albumin (17.3%). Approximately one-quarter of the patients exhibited myocardial injury with elevated levels of lactate dehydrogenase (LDH: 52.4%), myoglobin (MYO: 27.1%), cardiac troponin I (CTnI: 35.6%), N-terminal pro-B-type natriuretic peptide (ProBNP: 23.5%), and CK-MB (27.3%). Some patients exhibited kidney injury as indicated by elevated plasma urea (30.5%) and serum creatinine (10.2%) levels. Some patients also showed increased high sensitivity C-reactive protein (CRP: 54.4%) and procalcitonin (PCT: 32.6%) levels. In addition, some patients showed coagulation dysfunction, with elevated prothrombin time (PT: 17.9%), activated partial thromboplastin time (APTT: 14.7%), d-dimer (27.2%), and fibrinogen (Fbg: 21.3%).

**Table 3:** Initial laboratory indices of patients with COVID-19.

Variables	Normal range	No. of patients	Median (IQR)	No. of patients with value deviation (%)
Hematologic *10^**9**^**/L**				
White blood cells	3.5–9.5	197	5.78 (4.56–9.13)	68 (34.5%)^a^
Neutrophils	1.8–6.3	197	3.94 (2.48–8.14)	62 (31.5%)^a^
Lymphocytes	1.1–3.2	197	1.17 (0.71–1.78)	92 (46.7%)^b^
Platelets	125–350	197	197 (156–266)	37 (18.8%)^b^
CD3	723–2737	162	812 (395–1128)	77 (47.5%)^b^
CD4	404–1612	177	427 (190–615)	83 (46.9%)^b^
CD8	220–1129	173	291 (151–460)	66 (38.2%)^b^
CD16 + CD56	80–610	153	154 (117–251)	23 (15.0%)^b^
CD19	84–724	167	154 (105–254)	66 (39.5%)^b^
**Biochemical**				
AST, U/L	15–40	197	24 (16–38)	67 (34.0%)^a^
ALT, U/L	9–50	197	27(18–55)	72 (36.5%)^a^
ALP, U/L	10–60	197	58 (48.4–79)	68 (34.5%)^a^
Total bilirubin, μmol/L	0–23	197	8.9 (6.8–11.65)	15 (7.6%)^a^
Direct bilirubin μmol/L	0–8	197	4.6 (4–7.4)	24 (12.2%)^a^
Albumin, g/L	40.0–55.0	197	37 (32.9–39.5)	34 (17.3%)^a^
Potassium, mmol/L	3.5–5.5	191	140 (138–142)	91 (47.6%)^a^
Sodium, mmol/L	135–145	191	3.89 (3.6–4.37)	51 (26.7%)^a^
LDH, U/L	125–243	189	246 (172–375)	99 (52.4%)^a^
MYO, μg/L	0–100	192	56.6 (33.8–113)	52 (27.1%)^a^
CTnI, ng/mL	0–0.04	146	0.016 (0.006–0.08)	52 (35.6%)^a^
ProBNP		149	106.5 (38.1–551)	35 (23.5%)^a^
CK-MB, U/L	< 25	176	1.7 (1–5.21)	48 (27.3%)^a^
BUN, mmol/L	3.1–8	197	4.63 (3.95–7.35)	60 (30.5%)^a^
Creatinine, μmol/L	57–97	197	55 (49–99)	20 (10.2%)^a^
PT, s	9.4–12.5	195	11.8 (11.1–12.5)	35 (17.9%)^a^
APTT, s	25.1–36.5	190	28.9 (26.3–31)	28 (24.1%)^a^
d-dimer, mg/mL	0–0.55	195	0.8 (0.3–6.12)	53 (27.2%)^a^
Fbg, g/L	2–4	183	3.33 (2.68–4.83)	39 (21.3%)^a^
**Inflammation immunologic related indices**				
PCT, mg/L	< 0.05	184	0.09 (0.04–1.44)	60 (32.6%)^a^
CRP, mg/L	0–10	169	14.1 (5–73)	92 (54.4%)^a^
Serum ferritin, ng/mL	< 300	197	276 (230–445)	67 (34.0%)^a^
Procalcitonin, ng/mL	< 0.1	174	0.34 (0.06–1.49)	60 (34.5%)^a^
IL-6, pg/L	≤ 20	197	18.6 (14.5–27)	71 (36.0%)^a^

^a^Above reference. ^b^Below reference. Data are median (IQR), n (%).

There were many differences with regard to laboratory parameters between the non-aggravation and aggravation patients, as shown in Table 4. In the aggravation group, the number of neutrophils was notably increased (P < 0.001). Additionally, the total lymphocyte count was significantly decreased (P = 0.001). Subsequently, the cell count of the lymphocyte subtype was further analysed, which revealed that the counts of CD3^+^, CD4^+^, and CD8^+^ T cells in the aggravation group were significantly lower than those of the non-aggravation group (P < 0.05), but there was no difference in the counts of CD16^+^CD56^+^ T (P = 0.13) and CD19^+^ B cells (P = 0.06). Blood biochemical examination results suggested that patients with disease aggravation demonstrated higher levels of AST, ALT, DBIL, BUN, creatinine, CK-MB, CTnI, ProBNP, and LDH. Table 4 also revealed the differences in inflammation immunological-related indices between the two groups. This result suggested that the levels of CRP, serum ferritin, procalcitonin, and IL-6 were significantly higher in the aggravation group. At present, some researchers have reported that coagulation function is dysregulated with increased aggravation of the disease. We concluded that the levels of d-dimer and Fbg were markedly elevated in the aggravation patients.

**Table 4:** Laboratory findings of patients with and without disease progression.

Variables	Non-aggravation (n = 109)	Aggravation (n = 88)	p value
Hematologic *10^**9**^**/L**			
White blood cells	5.47 (3.56–7.18)	6.8 (4.44–13.13)	0.06
Neutrophils	2.81 (2.31–4.06)	7.75 (4.32–11.77)	< 0.001
Lymphocytes	1.56 (1.12–1.96)	0.755 (0.5–1.12)	0.001
Platelets	229 (159–266)	192 (150–218)	0.014
CD3^+^	985 (812–1311)	395 (113–641)	0.001
CD4^+^	558 (400–804)	235 (198–401)	0.001
CD8^+^	378 (274–526)	191 (73–307)	< 0.001
CD16^+^CD56^+^	104 (70–196)	98 (58–183)	0.13
CD19^+^	152 (117–251)	136(76–229)	0.06
**Biochemical**			
AST, U/L	22 (15–33)	35 (21–48.5)	0.01
ALT, U/L	26 (13–54)	36 (23–55)	0.04
ALP, U/L	55.7 (45–73)	58 (48.4–73)	0.78
Total bilirubin, μmol/L	9 (6.6–11.3)	8.6(7–13.79)	0.32
Direct bilirubin μmol/L	4 (3.9–5)	7.8 (4.6–11.4)	0.001
Albumin, g/L	38.8 (37–41.8)	35.9 (30.1–34.4)	0.16
BUN, mmol/L	4.04 (3.92–4.89)	7.82 (4.64–11.4)	0.001
Creatinine, μmol/L	52 (49–72)	69 (45.3–120.8)	0.04
Creatinine kinase, μM	60 (39–89)	69 (54.5–158)	0.06
CK-MB, U/L	1.02 (0.48–3.6)	2.5 (1.58–6.92)	0.001
CTnI, ng/mL	0.006(0.005–0.03)	0.034 (0.015–0.426)	0.001
ProBNP	38.1 (30.5–234)	209 (44–921)	< 0.001
LDH, U/L	199 (161–253)	355 (264–589)	0.002
Potassium, mmol/L	116 (103–140)	121 (88–140)	0.31
Sodium, mmol/L	3.94 (3.7–4.63)	3.7 (3.3–4.27)	0.07
**Coagulation function**			
PT, s	11.2 (11–11.9)	12 (11.7–13.4)	0.06
APTT, s	27.9 (25.4–30.5)	29.05 (26.9–32)	0.12
d-dimer, mg/mL	0.32 (0.15–0.8)	6.03 (0.9–18.6)	0.001
Fbg, g/L	2.87 (2.18–4.3)	3.63 (3.0–4.94)	0.001
**Inflammation immunologic related indices**			
CRP, mg/L	5 (1–15.5)	26.6 (10.7–122)	0.001
Serum ferritin, ng/mL	258 (208–303)	288 (254–699)	0.001
Procalcitonin, ng/mL	0.037 (0.027–0.06)	1.06 (0.11–3.26)	0.01
IL-6, pg/L	16.7 (14.4–20.4)	25 (17.6–64.5)	0.002

Data are median (IQR), n (%).

### Chest imaging features and pathogen examination

**Figure 2:**
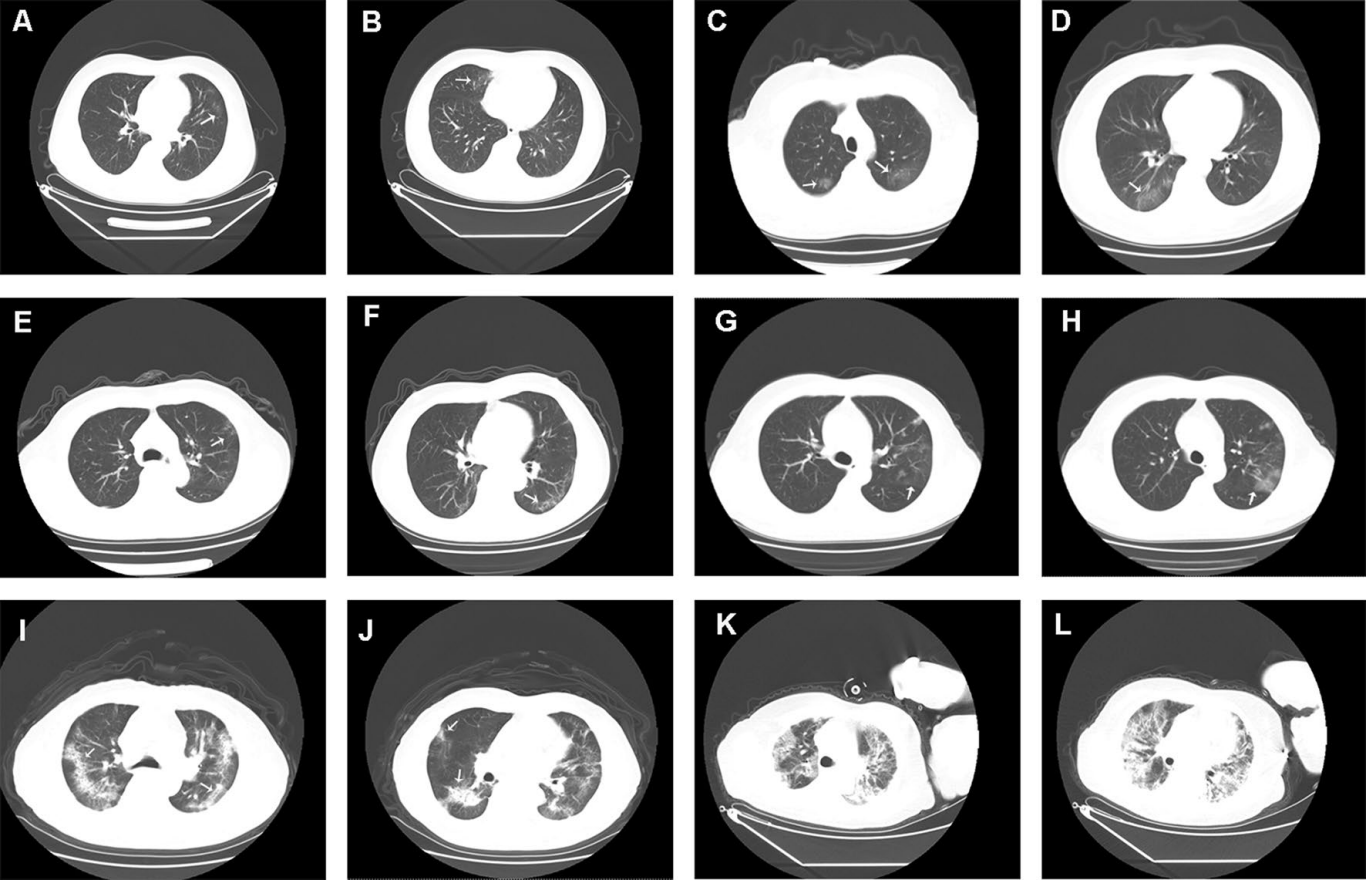
Chest CT images of the patients with COVID-19. (**A**,**B**) Images of a 38-year-old patient (Non-aggravation group) with few flocculent/patchy compact shadows in right lower lobe and left middle lobe. (**C**,**D**) Images of a 32-year-old patient (Non-aggravation group) with GGO in right middle/lower lobe and left middle lobe. (**E**,**F**) Images of a 58-year-old patient with multiple GGO, and patchy compact shadows at the periphery of the lung. (**G**,**H**) Images of a 69-year-old patient (Non-aggravation group) with GGO predominantly peripheral distribution in both lungs. (**I**,**J**) Images of an 89-year-old patient (Aggravation group) with diffuse, plaque-like, ground-glass opacities in both lungs centrally and peripherally. (**K**,**L**) Images of a 70-year-old patient (Aggravation group) with multiple typical GGO and multifocal consolidation shadows in bilateral lungs.

The chest imaging features upon admission are summarized in Table 5. The median time interval between symptom onset and CT examination was 5 days. Of the 197 patients, the majority (164, 83.2%) of the patients showed abnormal results: 107 cases (65.2%) had multiple ground-glass opacities, and 115 cases (70.1%) had bilateral lobular or subsegmental consolidation areas. A total of 62.8% of patients showed diffuse infiltration, and 42.4% had patchy shadows with interstitial involvement. Compared with the patients without aggravation, the aggravation group showed more bilateral ground-glass opacities and subsegmental areas of consolidation, as well as increased lung interstitial involvement. Most of the lesions were localized in the periphery of the lung. In the non-aggravation patients, the lesions were more localized in the periphery rather than in the centre of the lung (Fig. 2). The lesions had spread to the centre of the bronchus and gradually spread to the entire lung in aggravated patients. The SARS-CoV-2 PCR assay demonstrated that 167 (84.8%) cases showed positive results, and 30 (15.2%) cases showed negative results at the time of the first detection. In addition to SARS-CoV-2, we also detected other pathogens within the same patients, including Epstein Barr virus (EBV: 22 cases, 13.90%), *Mycoplasma pneumonia* (32 cases, 16.2%), influenza B virus (28 cases, 14.2%), parainfluenza virus (17 cases, 8.6%), and cytomegalovirus (CMV: 15 cases, 7.6%). There was no significant difference in the incidences of these pathogens between the disease aggravation group and the control group.

**Table 5:** Radiological data and pathogens test of the patients.

Clinical characteristics	Total patients	Non-aggravation	Aggravation	P
**Chest imaging features**	164 (83.2%)	87 (79.8%)	77 (87.5%)	
Ground-glass opacity	107 (65.2%)	55 (63.2%)	52 (67.5%)	0.56
Consolidation	115 (70.1%)	62 (71.3%)	53 (68.8%)	0.73
Bilateral infiltration	103 (62.8%)	47 (54.0%)	56 (72.7%)	0.01
Interstitial involvement	61 (37.2%)	31 (35.6%)	29 (37.7%)	0.79
**Lesion location**				
Peripheral	87 (53.0%)	59 (67.8%)	28(36.4%)	0.002
Central	15 (9.1%)	7 (8.0%)	8 (10.4%)	
Both peripheral and central	63 (38.4%)	22 (25.3%)	41 (53.2%)	
**Pathogens test**				
SARS-CoV-2 PCR assay+	167 (84.8%)	93 (85.3%)	74 (84.1%)	0.81
SARS-CoV-2 PCR assay±	30 (15.2%)	16 (14.7%)	14 (15.9%)	
EBV	22 (11.2%)	15 (13.8%)	7 (8.0%)	0.20
Mycoplasma pneumonia	32 (16.2%)	23 (21.1%)	9 (10.2%)	0.04
Influenza B virus	28 (14.2%)	15 (13.8%)	13(14.8%)	0.84
Parainfluenza virus	17 (8.6%)	9 (8.3%)	8 (9.1%)	0.84
CMV	15 (7.6%)	8 (7.3%)	7 (8.0%)	0.87

Data are n (%).

**Figure 3:**
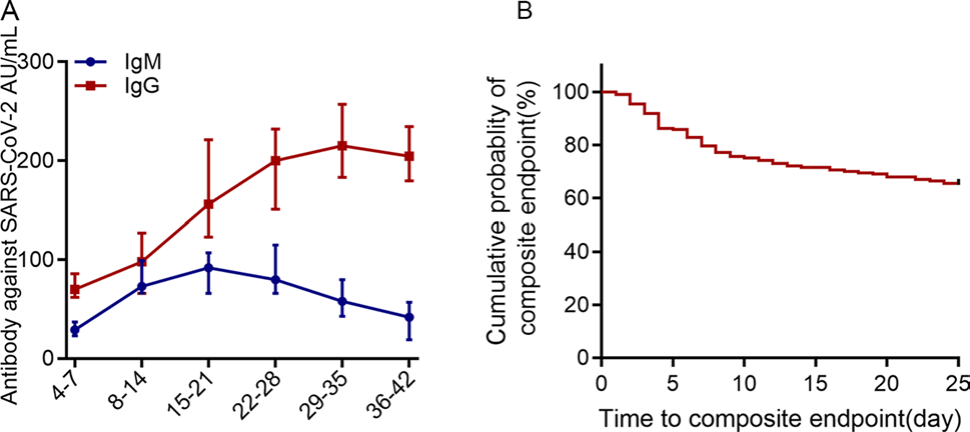
(**A**) The dynamic characteristics of IgM-IgG antibody levels. (**B**) Evaluation of the prognosis of COVID-19 patients via a Kaplan–Meier analysis. The composite endpoint (event) was ICU transfer or death within 25 days from the date of admission to the hospital. The cumulative event-free survival curve was plotted.

Nucleic acid testing is the standard method for the diagnosis of COVID-19 infections^6^. However, some research has suggested that this method usually shows lower positive rates due to poor RNA stability, different specimen positions, and quality^7^. Therefore, the IgM-IgG combined assay has been recommended to increase the sensitivity of COVID-19 diagnoses, especially in patients with suspected COVID-19 infections^8^. In this study, dynamic changes in IgM-IgG antibody levels were detected. Specific IgG and IgM antibodies can be detected 4–7 days after the onset of the illness. The IgM antibody titres sharply and significantly increased in the initial 2 weeks, peaked at 1–2 weeks after symptom onset, and significantly declined after 21 days. The IgG antibody titres increased over time, peaking at 4–5 weeks after the onset of the illness, after which higher levels were maintained for the entire observation period (Fig. 3A). In addition, we compared the IgM-IgG levels between the two groups and found that there was no difference in the levels of IgM between the aggravation group and the non-aggravation group during the first 2 weeks. After this time interval, the aggravation group tended to have a more vigorous IgM response against SARS-CoV-2 and displayed a higher peak, which suggested that serum IgM antibody levels were significantly correlated with disease aggravation from 3 to 4 week onward. However, the levels of IgG in the aggravation group were markedly lower than those in the non-aggravation group in the early stages of the infection, after which the levels exhibited significant increases rapid growth that exceeded those of the non-aggravation group.

### Treatments and clinical outcomes

All of the confirmed patients were isolated and treated in a negative pressure ward. Table 6 includes details of the treatments and outcomes of the COVID-19 infection patients. Most patients received combination therapy with antibiotics, antiviral agents, oxygen support, and glucocorticoids. 101 (51.3%) patients received antibiotics treatments (cephalosporins, quinolones, carbapenems, tigecycline). More than one intravenous antibiotic was given to 65 (33.0%) patients and 87 (44.2%) received an antibiotic only. 145 (73.6%) patients were given antiviral treatment including ganciclovir, oseltamivir, ritonavir, and lopinavir. Among the 87 (44.2%) patients who required systemic glucocorticoid therapy (methylprednisolone, dexamethasone) over the treatment period, 49 (24.9%) patients were given low-dose glucocorticoids and 38 (19.3%) were given standard-dose glucocorticoids at the initiation of treatment. 121 (61.4%) received Chinese traditional medicine treatment, which has been demonstrated to play an important role in resistance to viral infections^9,10^. These Chinese traditional medicines were generally used in China during COVID-19 outbreak, like Astragali Radix (Huangqi), Atractylodis Macrocephalae Rhizoma (Baizhu), Glycyrrhizae Radix Et Rhizoma (Gancao), Saposhnikoviae Radix (Fangfeng)^11–13^. Of the 197 patients, more than two-thirds of the patients required oxygen therapy. A total of 105 (53.3%) patients were treated with high-flow oxygen. Forty-three (21.8%) patients were given non-invasive mechanical ventilation. Twenty-five (12.7%) patients received invasive mechanical ventilation. The study also indicated that antibiotics, corticosteroids, and oxygen therapy were necessary more often in aggravation patients than in non-aggravation patients (P < 0.05).

**Table 6:** Treatment and outcome of COVID-19 patients.

Treatments	Total	Non-aggravation	Aggravation	p value
Antibiotics (n/%)	101 (51.3%)	22 (20.2%)	79 (89.8%)	< 0.001
Antiviral (n/%)	145 (73.6%)	81 (74.3%)	64 (72.7%)	0.80
Corticosteroids (n/%)	87 (43.7%)	19 (17.4%)	68 (77.3%)	< 0.001
Chinese medicine (n/%)	121 (61.4%)	64 (58.7%)	57 (64.8%)	0.39
Oxygen treatment (n/%)	134 (68.0%)	46 (42.2%)	88 (100%)	< 0.001
High-flow oxygen therapy (n/%)	105 (53.3%)	38 (34.9%)	67 (76.1%)	< 0.001
Noninvasive mechanical ventilation (n/%)	48 (24.4%)	6(5.5%)	42 (47.7%)	< 0.001
Invasive mechanical ventilation	25 (12.7%)	4 (3.7%)	21 (23.9%)	< 0.001
**Outcomes (n/%)**				
ARDS	48 (24.4%)	10 (9.2%)	38 (43.2%)	< 0.001
Septic shock	29 (14.7%)	9 (8.3%)	20 (22.7%)	0.004
DIC	17 (8.6%)	4 (3.7%)	13 (14.8%)	0.005
Fungal infections	27 (13.7%)	11 (10.1%)	16 (21.9%)	0.10
Acute cardiac injury	32 (18%)	11 (10.1%)	21 (22.7%)	0.009
Acute kidney injury	25 (12.7%)	5 (4.6%)	20 (22.7%)	<0.001
ICU admission	60 (30.5%)	11 (10.1%)	49 (55.7%)	< 0.001
Length of ICU stay (d)	7 (2–13)	5 (2–8)	8 (2–13)	0.15
Length of hospital stay (d)	15 (9–21)	11 (9–19)	18 (13–23)	0.003
Duration of viral shedding after onset (d)	11(6.5–17)	9(6–14)	13(8–18)	0.04

Data are median (IQR), n (%).

By the end of March 15, 164 (83.2%) patients had improved and were discharged. The median length of stay of the patients was 15 days (IQR: 9–21). Of the 88 aggravation patients, 49 (55.7%) patients were admitted to the ICU for a median of 8 days, which were significantly higher than that of the non-aggravation patients for a median of 5 days. To evaluate the prognoses of the COVID-19 patients, we performed a Kaplan–Meier analysis, and the composite endpoint (event) was ICU transfer or death within 25 days from the date of admission to the hospital. The cumulative event-free survival curve is plotted in Fig. 3B, which suggests that 64.9% of the patients had not experienced ICU transfer or survival from the hospital. During the course of the disease, approximately 103 (52.3%) patients presented with functional damage involving multiple vital organs, including ARDS (48 cases, 24.4%), septic shock (29 cases, 14.7%), DIC (17 cases, 8.6%), fungal infections (27 cases, 13.7%), acute cardiac injury (32 cases, 18.0%), and acute kidney injury (25 cases, 12.7%). Patients with disease aggravation showed higher complication rates than patients without disease aggravation (Table 6).

**Table 7:** Bivariate cox regression of factors associated with disease progression of COVID-19.

Variables	Univariate analysis			Multivariate analysis		
	OR	95% CI	P	OR	95% CI	P
Age (≥ 60 years vs. < 60 years)	4.17	2.30–7.59	< 0.001	2.74	1.03–7.27	**0.04**
Gender (male vs female)	3.0	1.67–5.70	< 0.001	1.03	0.99–1.07	0.09
**Comorbidity (yes vs no)**						
Hypertension	2.17	1.14–4.06	0.02	3.64	1.28–10.3	**0.02**
Diabetes	3.0	1.48–6.38	0.002	8.31	2.92–23.6	**0.001**
**Signs and symptoms (yes vs no)**						
Dyspnea	2.01	1.14–3.62	0.018	6.17	2.01–18.9	**0.001**
Chest distress	3.0	1.48–5.86	0.001	1.85	0.76–4.5	0.18
**Hematologic**						
Neutrophils	6.83	3.53–17.6	< 0.001	0.99	0.96–1.01	0.41
Lymphocytes	0.12	0.06–0.22	< 0.001	0.29	0.10–0.86	**0.02**
CD3	0.08	0.04–0.17	0.002	0.101	0.01–1.78	0.12
CD4	0.74	0.59–0.93	0.01	0.88	0.21–3.7	0.86
CD8	0.25	0.13–0.49	0.03	1.01	0.99–1.03	0.16
**Coagulation function**						
Fbg	4.35	2.33–8.14	0.002	9.72	2.6–36.4	**0.001**
d-dimer	9.59	4.48–20.51	< 0.001	1.75	0.37–8.24	0.48
**Biochemical**						
AST	2.09	1.11–3.93	0.03	0.99	0.93–1.07	0.98
BUN	5.17	2.66–10.0	0.001	1.19	0.96–1.46	0.11
Creatinine kinase	11.67	2.58–52.80	0.001	1.09	0.96–1.24	0.19
LDH	8.6	4.43–16.69	0.001	1.01	0.95–1.02	0.06
CTnI	4.78	2.15–10.6	0.002	10.06	2.44–41.2	**0.001**
ProBNP	5.89	3.64–9.04	0.001	0.10	0.93–1.07	0.98
**Infection-related indices**						
IL-6	3.26	1.77–5.99	0.001	1.03	1.01–1.06	**0.02**
CRP	5.99	1.18–30.30	0.03	1.01	0.99–1.03	0.33
PCT	3.73	1.96–7.12	0.001	2.11	0.67–6.62	0.20
Serum ferritin	2.30	1.26–4.20	0.006	1.01	1.0–1.02	**0.04**

Bold means the data are statistically significant.

### Risk factors for disease aggravation

To investigate the potential risk factors for disease aggravation, we compared the epidemiological and clinical characteristics of COVID-19 patients between the two groups. In the univariate logistic regression analysis, we found that older age, male sex, underlying diseases (hypertension and diabetes), dyspnoea, and chest tightness were associated with a higher risk of disease aggravation. Lymphopenia, neutrophilia, and high levels of AST, BUN, CK, LDH, CTnI, ProBNP, Fbg, d-dimer, IL-6, CRP, procalcitonin, and serum ferritin were all significantly correlated with disease aggravation. Subsequently, these variables were included in the multivariable logistic regression model, which indicated that older age, hypertension, diabetes, dyspnoea, lymphocytes, Fbg, CTnI, IL-6, and serum ferritin were all independently associated with disease aggravation (Table 7).

## Discussion

The present study included a total of 197 patients who were hospitalized with COVID-19 from January 25, 2020, to March 15, 2020. All of the patients were evaluated for therapeutic efficacy after at least 1 week of hospitalization. The results identified disease aggravation in 88 patients and non-aggravation of the disease in 109 patients. We summarized the clinical characteristics and identified several risk factors that were associated with disease aggravation in the patients. As of March 15, 2020, 164 (83.2%) patients improved and were discharged, and 33 (16.8%) patients died. Although all of the age groups have been affected by COVID-19, elderly patients (over 60-years-old) have a greater risk for infection and a relatively high proportion of severe and disease aggravation cases^14–17^. These results showed that advanced age is an independent risk factor for disease progression. Growing evidence demonstrates that males were more susceptible to acquire viral infections and disease progression. A study from China firstly reported the presence of gender differences which suggested that males account for 60% of the COVID-19 patients^18^. And then a study examining 799 patients found that 73% of patients who died (113 patients) were male. Another study suggested that male was a significant risk factor for disease progression^19^. According to the last sex-related study conducted in Italian on 239,709 patients, lethality is more prevalent in males compared to females (17.7% vs 10.8%), and males account for 59% of deaths from COVID-19^20^. Further, a system review with 59,254 patients from 11 different countries demonstrated that males had much higher mortality risk than females^21^. In the present study, we found that males were identified more likely to be contaminated with the virus (60.9% vs 39.1%), and had inferior outcomes than the female patients. Therefore, our study further confirmed that males were more susceptible to acquire viral infections and disease progression.

Of all of the patients, 72 (36.5%) patients cases had one or more underlying diseases, including diabetes, hypertension, and COPD. The logistic regression analysis confirmed a higher frequency of hypertension and diabetes in patients with disease aggravation. It has been reported that patients with hypertension or diabetes accounted for 20–30% of the total infected patients and comprised over half of the patients in the ICU^22,23^. Recently, a retrospective cohort study also demonstrated that these comorbidities have been responsible for 60.9% of deaths caused by COVID-19^24^. A large number of studies have indicated that the angiotensin-converting enzyme 2 (ACE2) receptor is highly expressed in the cardiovascular/cerebrovascular and lung tissues in hypertension patients^25^. In addition, ACE2 is one of the most important host receptors of H7N9, SARS, and COVID-19, whose activities are closely related to the pathogenesis of inflammatory and acute injuries of lung disease caused by these viruses^25–27^. Experimental studies have suggested that the spikes of SARS-CoV-2 could bind to ACE2 receptors, which can mediate virus entry into HeLa cells^24,28^. Some scholars have found that most COVID-19 patients showed higher angiotensin II activity than uninfected people, and the abnormal increase in angiotensin II was related to lung failure and death^29^. It has been established that the expression of ACE2 is significantly upregulated in COPD and in cigarette-smoking patients^30^. Several studies have demonstrated that smokers and patients with COPD have a high rate of SARS-CoV-2 infection and increased risks of severe complications and mortality^31^. A meta-analysis by Carlos A. J. included 6487 cases from 19 studies and revealed that smoking status could produce a more serious clinical condition of COVID-19 and can more likely lead to ICU admission and death^32^. However, several studies have shown that smoking was not associated with an increased risk of critical illness^23,33^. In the current study, we found that smoking was not associated with disease aggravation or critical illness. This may be explained by the small number of available studies, small sample sizes, and short lengths of study. ACEIs and ARBs are recommended for the management of hypertension and renal disease associated with diabetes^34^. Recent studies have reported that the application of ACE inhibitors can induce a marked increase in ACE2 expression, which means that ACEIs/ARBs would increase the risk of COVID-19 infection and disease aggravation of COVID-19 in hypertension and diabetes patients receiving these drugs^35,36^. In the present study, COVID-19 patients with ACEI/ARB therapy were enrolled, and we found that there were more patients taking ACEIs/ARBs in the aggravation group. Therefore, taking ACEIs/ARBs may be another potential risk factor for the disease aggravation of COVID-19.

In our cohort, no significant difference in the median days from symptom onset to hospital admission was found between the disease aggravation and non-aggravation patients. Consistent with the symptoms that have been previously reported^22^, the most common symptoms were fever, cough, fatigue, chest tightness, and myalgia or arthralgia. The proportions of patients with myalgia or arthralgia, chest distress, and dyspnoea were significantly higher in the disease aggravation group. Remarkably, few patients exhibited some of the less common symptoms, including abdominal pain, vomiting, and conjunctival hyperaemia, which may result in missed diagnoses and transmissions to other people. Previous studies have found that the virus could be detected in stool samples in patients with symptoms of abdominal pain and vomiting, as well as in asymptomatic patients^37,38^. Therefore, further research is still required to determine whether faecal oral transmission exists.

SARS-CoV-2-induced immune responses and infection cytokine storms are believed to play major roles in disease aggravation. The destruction of lung cells can recruit macrophages and monocytes and release cytokines to resolve the infection and even simultaneously mediate widespread excessive inflammation^39,40^. In the present study, 92 (46.7%) patients showed significant neutrophilia and lymphopenia with pronounced reductions in peripheral blood CD3^+^, CD4^+^, and CD8^+^ cells. Lymphocyte subsets, especially CD3^+^, CD4^+^, and CD8^+^ cells, were more severely impaired in disease aggravation patients. This is consistent with a previous study by Qin et al*.*, which demonstrated that the percentages of memory, as well as the percentages of regulatory and effector T cells, were significantly decreased in severe cases, compared to non-severe cases^39,41^. Furthermore, we found that CD19^+^ cell counts also exhibited a decreasing trend, compared with matched controls, but this difference did not reach statistical significance. These results were consistent with the study conducted in Spain, which demonstrated that there were no differences in CD19^+^ B cell percentages between the survivor and non-survivor groups^42^. The plausible explanation for this discrepancy may be due to the direct infection of T lymphocytes by SARS-CoV-2, cytokine-mediated lymphocyte trafficking into infected tissues, or lymphocyte exhaustion in the peripheral blood^42,43^. All of these hypotheses remain to be proven in further investigations.

Previous studies have shown that serum inflammation-related indices are closely related to the degree of inflammation and disease severity^40^. In this study, compared with patients without disease aggravation, the disease aggravation patients showed significantly increased expression of inflammation-related factors, including IL-6, CRP, serum ferritin, and procalcitonin levels. Multivariate analysis revealed that elevated levels of IL-6 and serum ferritin were risk factors for disease aggravation in the patients. It has been previously established that patients with severe SARS and MERS have a higher incidence of multiple organ dysfunction syndromes, including liver damage, acute heart/kidney injury, coagulation dysfunction, and even death^44,45^. Many studies have demonstrated the clinical characteristics and laboratory findings associated with different degrees of multiple organ dysfunction in patients with COVID-19^16,46^. In our study, liver damage and acute heart/kidney injury were considered to occur in more than one-third of patients. The levels of AST, ALT, direct bilirubin, LDH, creatinine, BUN, CTnI, and ProBNP seemed to be significantly higher in patients with disease aggravation. The multivariate analysis revealed that elevated levels of LDH, CTnI, and ProBNP were risk factors for disease aggravation. Currently, there are three potential mechanisms for this observation. First, the binding of SARS-CoV-2 to ACE2-positive cells mediates direct damage. Second, systemic inflammatory response syndrome includes a cytokine storm, dysregulated immunocytes, and uncontrolled inflammation. Third, exogenous drugs induce organ metabolizing burden or worsen organ function^47,48^. Recently, coagulation dysfunction has attracted increased attention among scholars. It has been reported that the incidence of coagulopathy in all patients showed abnormalities of varying degrees. A previous study revealed that Fbg and d-dimer elevations were related to the severity of the disease^49^. Ji et al. demonstrated that coagulation activation and hyperfibrinolysis coexisted in patients with severe COVID-19 infections^50^. Patients with elevated plasmin and Fbg levels may have an increased risk of ARDS and mortality^51^. In this study, we found that coagulation function parameters (APTT, Fbg, and d-dimer) were higher in patients with disease aggravation. A further analysis revealed that a high level of Fbg was significantly associated with the outcome. Therefore, measurements of these coagulation function parameters may be important biomarkers of disease aggravation and outcomes.

Currently available treatment approaches for COVID-19 include symptomatic and supportive therapies, such as oxygen therapy, antivirals, prevention and treatment of infections, and combination treatment with glucocorticoids^52,53^. In our study, most patients received combination therapy with oxygen support, antibiotics, antiviral agents, and glucocorticoids. Several antiviral agents previously used to treat SARS and Middle East Respiratory Syndrome (MERS) and HIV have been considered as the most potential candidates for COVID-19 patients. With the spread of COVID-19, a large number of clinical studies all over the world have been underway to investigate the potential agents for COVID-19. Some preclinical research has suggested that remdesivir has broad-spectrum antiviral activity against MERS-CoV and SARS-CoV-1/2 infections^54^. Several studies indicated that COVID-19 patients would benefit from the combination of lopinavir and ritonavir with fewer adverse clinical outcomes^55,56^. However, further studies on a larger set of clinical specimens will be required to assess the efficacy and safety of antiviral drugs^55,57,58^. It has been reported that coinfection with bacteria has a great influence on the disease progression and prognosis of COVID-19 patients, which may lead to increased needs for ICU care, and increased mortality rate especially in severe patients^59,60^. Brockmeier et al. reported that coinfection with bacteria could significantly upregulate the levels of inflammatory cytokines, especially IL-6 and MCP-1 in COVID-19 patients^61^. Martins- Filho et al. demonstrated that the bacterial and fungal coinfection had an increased mortality by 2.5 folds in COVID-19 patients^62^. Therefore, antibiotic treatment for COVID-19 patients based on the experience of overlapping bacterial infections and results of antimicrobial susceptibility test seemed to be the basic principle. In our study, 101 (51.3%) patients received antibiotics treatments, and the aggravation patients were more likely to receive antibiotics than the non-aggravation patients. Due to the cytokine storm, corticosteroids are widely used in the treatment of patients with severe illness to attenuate inflammation that is associated with lung injury. In the present study, the disease aggravation patients were significantly more likely to receive glucocorticoid therapy. Nevertheless, some researchers have stated that the use of glucocorticoids did not reduce mortality but could easily lead to disease aggravation and increase the risk of secondary infections^63^. Therefore, the rational use of an appropriate dose of glucocorticoids could suppress the excessive activation of the immune system and the secretion of inflammatory cytokines. Almost all of the patients received respiratory support, including nasal cannula oxygen and continuous positive air pressure. The aggravation group was significantly more likely to receive higher levels of respiratory support. Undoubtedly, patients with disease aggravation have a higher mortality rate than patients without disease aggravation. Additionally, ARDS remained to be the most common cause of death, followed by multiple organ failure. Therefore, the prevention and treatment of ARDS represent an important strategic objective for the reduction of mortality and morbidity.

## Limitations

This study also has severe limitations. First, the inherent shortcomings are attributed to a retrospective observational study, and the small sample size and short-term follow-up make it difficult to obtain a firm conclusion. Second, the present study had possible patient selection bias. Therefore, a larger cohort study of patients from China and other countries may help to further investigate the clinical characteristics and risk factors for the outcomes. Finally, the actual duration of viral clearance was overrated, which is due to the frequency of respiratory specimen collection. In addition, the viral load of SARS-CoV-2 was not accurately quantified, and false-negative results for upper respiratory samples have also been reported. Therefore, studies on the dynamic characteristics of the viral load are still warranted.

## Conclusions

Investigating the potential factors of advanced age, comorbidities and elevated level of Fbg, CTnI, IL-6, serum ferritin enables early identification and management of patients with poor prognosis. In addition, detection of the dynamic antibody may offer vital clinical information during the course of SARS-CoV-2 and provide prognostic value for infected patients. More related studies are needed in the future.

## Methods

### Study participants and design

This study was conducted in accordance with the Declaration of Helsinki and was approved by the Institutional Review Board of Taihe and Jinzhou central hospital. All of the patients were consecutively enrolled from January 18, 2020, to March 15, 2020, in Taihe and Jinzhou Central Hospital, and the patients had been confirmed to have COVID-19 infections according to World Health Organization interim guidance. All of the participants provided written informed consent before the data were retrospectively collected.

### Data collection

We reviewed the clinical medical records of all of the patients to collect the demographic data and clinical and laboratory findings of the patients. All of the data, including age, sex, occupation, onset of symptoms, underlying comorbidities, laboratory results, treatment, and outcome data, were retrospectively extracted with standardized data collection forms. The clinical outcomes were followed up to March 15, 2020.

To identify the COVID-19 cases, nasal or pharyngeal swab samples were obtained from the patients at admission and were tested via real-time reverse transcriptase polymerase chain reaction (RT-PCR). The exact date of onset of symptoms was defined as the day when the symptom was first noticed. A weekly assessment of disease aggravation was performed that provided details about the patients’ clinical statuses. All of the study participants were divided into either the disease aggravation group or the non-aggravation group, based on the following specific criteria: (1). aggravation group composed of patients who had a higher body temperature than before, aggravated symptoms, and a varied aggravation of imaging examination manifestation; and (2). non-aggravation group composed of patients who had decreased body temperature, symptom and imaging improvements, or no significant changes in body temperature, as well as respiratory symptoms and imaging manifestations. Smoking history was defined as the smoking of at least one cigarette per day and a smoking duration lasting for more than 1 year^64^. ARDS and shock were defined according to the interim guidance of the WHO for the novel coronavirus^65^. Acute kidney injury was identified and classified based on the highest serum creatinine level or urine output criteria according to the kidney disease improving global outcomes classification^66^. Cardiac injury was diagnosed if serum levels of cardiac biomarkers (e.g., troponin I) were above the 99th percentile upper reference limit or if new abnormalities were shown in the electrocardiography and echocardiography tests^67^. Finally, a total of 197 subjects were enrolled in the study and were divided into two groups: 88 cases with disease aggravation and 109 cases without disease aggravation. The flow chart of the study is presented in Fig. 1.

### Statistical analysis

The categorical variables were expressed as frequencies and numbers (%) and were analysed using the chi-squared test or the Fisher’s exact test. The continuous variables are presented as median or interquartile range (IQR) values, and two-sided unpaired t-tests or Mann–Whitney tests were used to compare the groups. All of the statistical analyses and graphs were generated and plotted by using GraphPad Prism (version 7.0) and SPSS (version 21.0). A confidence level of P < 0.05 was considered to be statistically significant for all of the analyses.
